# Balance in Virtual Reality: Effect of Age and Bilateral Vestibular Loss

**DOI:** 10.3389/fneur.2017.00005

**Published:** 2017-01-20

**Authors:** Elodie Chiarovano, Wei Wang, Stephen J. Rogers, Hamish G. MacDougall, Ian S. Curthoys, Catherine de Waele

**Affiliations:** ^1^School of Psychology, University of Sydney, Sydney, NSW, Australia; ^2^Cognition and Action Group, CNRS UMR8257, University of Paris Descartes, Paris, France; ^3^University of Hangzhou Dianzi, Hangzhou, China; ^4^Pitie Salpetriere Hospital, ENT Department, Paris, France

**Keywords:** Wii Balance Board, bilateral areflexia, iPod Touch, visual distractor, visual perturbation, foam rubber, BalanceRite, postural stability

## Abstract

**Background:**

Quantitative balance measurement is used in clinical practice to prevent falls. The conditions of the test were limited to eyes open, eyes closed, and sway-referenced vision. We developed a new visual perturbation to challenge balance using virtual reality (VR), measuring postural stability by a Wii Balance Board (WBB).

**Methods:**

In this study, we recorded balance performance of 116 healthy subjects and of 10 bilateral vestibular loss patients using VR to assess the effect of age and the effect of total loss of vestibular function. We used several conditions: eyes open (normal visual inputs), eyes closed (no visual inputs), stable visual world (vision referenced), and perturbed visual world (visual perturbation) at different amplitudes of perturbation. Balance under these visual conditions was assessed on the WBB (stable support surface) and on the WBB plus foam rubber (unstable support surface).

**Results:**

In healthy subjects, we found that the percentage of falls increased with age and with the amplitude of perturbation for both conditions: WBB or WBB + foam. Moreover, we can define a threshold for falls in each age group as the amplitude of perturbation which induced falls. For bilateral vestibular loss patients, on the WBB + foam, all of them failed with eyes closed and with perturbed visual world even at the minimal amplitude of perturbation. Finally, we observed that stable visual world induced fewer falls than eyes closed whatever the subject’s group (healthy or bilateral vestibular loss) and whatever the age decade.

**Conclusion:**

VR allowed us to develop a useful new tool with a wide range of visual perturbations. Rather than only two levels of visual condition (eyes open and eyes closed), the VR stimulus can be continuously adjusted to produce a visual perturbation powerful enough to induce falls even in young healthy subjects and which has allowed us to determine a threshold for falls.

## Introduction

Assessment of the balance performance of vestibular patients and senior subjects is an important part of clinical evaluation to prevent falls (1). Several methods of testing exist, from the Romberg to the Equitest (2). Recently, with the development of low-cost force platforms, the Wii Balance Board (WBB) is being used for balance testing (3). Two years ago, we developed an App called *BalanceRite*, which recorded the time series data from the WBB to an iPhone or iPod Touch. The validity of *BalanceRite* was established by comparing its results to the results of the Equitest (4). One year ago, we developed a visual perturbation using virtual reality (VR) to perturb visual inputs (4). The effectiveness of VR using different headsets has been shown to substantially perturb the stability of healthy subjects (5). We are able to challenge two sensory inputs involved in balance: visual inputs by using VR and proprioceptive inputs by using foam rubber on the WBB (WBB + foam). The WBB allows measurement of postural stability during visual or visual plus proprioceptive (foam) perturbation. Previously, postural stability in healthy young subjects was assessed by using only eyes open, eyes closed, or sway-referenced vision. Such conditions have been too easy, so the upper limit of stability was never reached (6). With the visual perturbation, we have a full range of perturbation from absolute stability to fall in perfectly healthy young subjects. To develop age-dependent normative data, here we report the results of using this effective visual perturbation on the balance performance of healthy subjects at increasing decade age bands and of patients with bilateral vestibular loss (BVL).

Our aims were threefold:

1. To study the effect of age on balance performance using a visual perturbation delivered by VR for healthy subjects.
2. To study the effect of complete vestibular loss on balance performance using VR.
3. To show the advantage of using the VR for the assessment of balance in contrast to simple “eyes closed” testing.

## Materials and Methods

All subjects included in the study gave written and informed consent. The study followed was in accordance with the ethical standards of the Helsinki Declaration and was approved by the University of Sydney Human Ethics Committee—Protocol number 2013/288.

One hundred sixteen healthy subjects (64 females/52 males, mean age: 58 ± 20 years, range: 20–89 years) and 10 BVL patients (2 females/8 males, mean age: 52 ± 11 years, range: 35–68 years) were tested for balance performance on the WBB and on the WBB plus foam (WBB + foam). The foam rubber used was an Airex Balance Pad blue (Airex AG, Sins, Switzerland, 41 cm × 50 cm × 6 cm thick). The condition’s order (WBB first or WBB + foam first) was randomized to minimize learning processes. Several visual conditions were assessed: eyes open, eyes closed, VR with a stable environment, and VR in perturbed world environment with several amplitudes of the perturbation. Each condition lasted for 25 s while subjects stood on the WBB with feet 7 cm apart.

The VR environment [previously described in Ref. (4)] was a moving visual scene of a house and garden, sky and sea (modified from the “Oculus Tuscany Demo” developed by FenixFire and Oculus VR, Irvine, CA, USA). With our custom program, this scene could be rotated around pitch, roll, or yaw axes, and during testing, the scene was unpredictably rotated by sum-of-sines pseudorandom waveforms, which drove each axis differently. To maximize the unpredictability of the motion of the world, we used different frequencies for rotations on the X, Y, and Z axis. The X axis rotation was the sum of three sine waves with frequencies of 0.5, 0.2, and 0.1 Hz and 0° phase. The Y axis rotation was the sum of three sine waves with frequencies of 0.4, 0.1, and 0.1 Hz and phase angles of 0°, 25°, and 0°, respectively. The Z axis rotation was the sum of three sine waves with frequencies of 0.5, 0.2, and 0.2 Hz and phase angles of 70°, 45°, and 90°. For each of these, it was possible to control the peak amplitude of the rotation (between 0° to 30°), which were arbitrary, reported on a VR scale between 0 and 1. In this experiment, the peak amplitude of the rotation was fixed at 0° (stable world, VR0), 3° (VR0.1), 6° (VR0.2), 9° (VR0.3), 12° (VR0.4), 15° (VR0.5, see Video S1 in Supplementary Material), or 30° (VR1.0).

**Figure 1:**
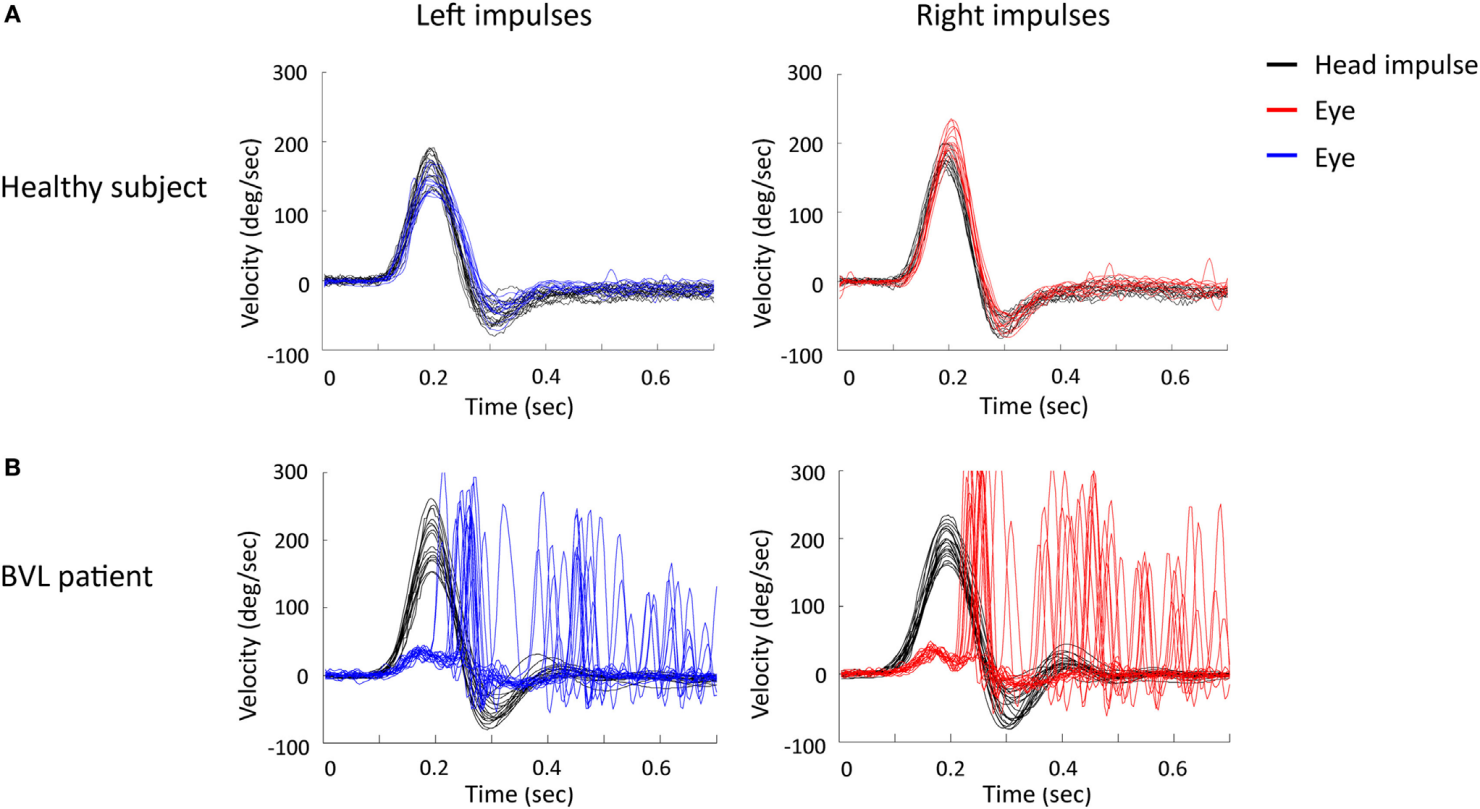
**Video head impulse test (vHIT) of a healthy subject (A) and a BVL patient (B) for horizontal head impulses (head velocity in black) to the left side (eye velocity in blue) and to the right side (eye velocity in red)**. Noted the vHIT of BVL patient showed a decreased gain and many catch-up saccades on both sides. BVL, bilateral vestibular loss; sec, seconds; deg, degrees.

Subjects were recorded at the Pitie-Salpetriere Hospital (Paris) between June 2015 and March 2016. Subjects with a history of vestibular or neurological disorders were excluded from the healthy group. Subjects were included in the healthy group even if they complained of hearing loss, tinnitus, or postural instability (seniors). The video head impulse test (vHIT) was systematically performed to verify normal vestibular canal function in healthy subjects (Figure 1A) and to confirm the complete vestibular loss in BVL patients (Figure 1B) (7). The distribution of healthy subjects per decade and the repartition into two groups (young controls and seniors) are given in Table 1. The percentage of falls for each condition for healthy subjects and for BVL patients is reported in the Section Results. A condition is defined as a fall if the subject failed two consecutive times in the condition. If a subject failed at the first attempt and succeeded at the second attempt, the condition is defined as a success and the next condition is assessed. To avoid any injury in case of a potential fall of the subject, every subject was tested with two experimenters (one on each side of the subject).

**Table 1:** **Social characteristics of healthy subjects per decade of age and segregated into two groups: young controls and seniors**.

Decades	Characteristics	Groups	Characteristics
20–29 years	N = 15 (8 F/7 M)	Young controls	N = 63 (35 F/28 M)Mean age: 43 ± 15 yearsRange: 20–65 years
30–39 years	N = 9 (5 F/4 M)		
40–49 years	N = 15 (8 F/7 M)		
50–59 years	N = 16 (8 F/8 M)		
60–69 years	N = 17 (13 F/4 M)		
		Seniors	N = 53 (29 F/24 M)Mean age: 76 ± 7 yearsRange: 66–89 years
70–79 years	N = 26 (13 F/13 M)		
80–89 years	N = 18 (9 F/9 M)		

*F, females; M, males*.

## Results

### On the WBB

**Figure 2:**
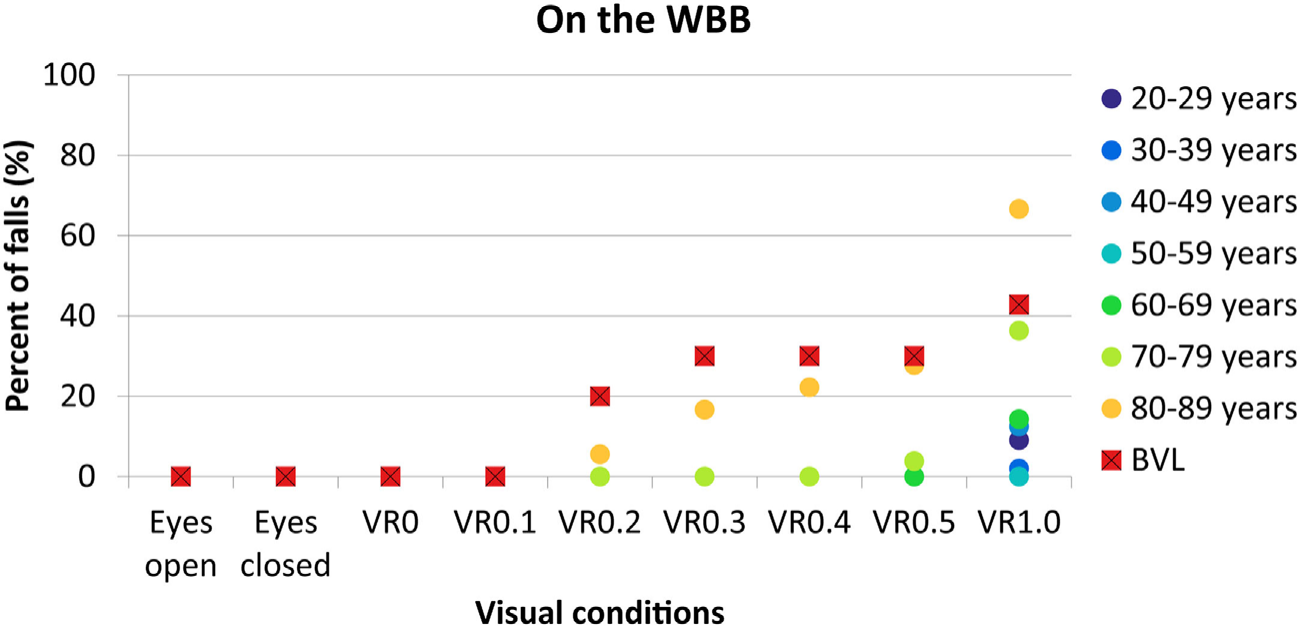
**Percentage of falls on the WBB in function of visual conditions for each decade of age from 20–29 years (dark blue circles) to 80–89 years (yellow circles) and for BVL patients (red squares with black cross)**. WBB, Wii Balance Board; VR, virtual reality; BVL, bilateral vestibular loss.

Percentages of falls per decade and for BVL patients on the WBB for each condition are given in Figure 2.

No falls were observed with eyes open, eyes closed, VR0 and VR0.1 for each age group or for BVL patients.

No falls or very few (1 subject, <4%) were observed with visual perturbations of VR0.2, VR0.3, VR0.4 and VR0.5 for healthy subjects between 20 and 79 years. Above 80 years, the percentage of falls increased with the amplitude of the perturbation: 5% with VR0.2, 16% with VR0.3, 22% with VR0.4, and 27% with VR0.5. For BVL patients, we observed 20% of falls with VR0.2 and 30% with VR0.3, VR0.4, and VR0.5. At VR0.5, percentages of falls in BVL group and in 80- to 89-year-old group were similar (difference non-statistically significant, exact Fisher test, *p* = 1).

Finally, at VR1.0, between 0 and 14% of falls are observed below 70 years. There were 36% of falls in the decade 70–79 years and 66% in the decade 80–89 years. In the BVL group, 42% of falls were observed, which is similar to the percentage of falls in the 70–79 decade (difference non-statistically significant, exact Fisher test, *p* = 1).

### On the WBB + Foam

**Figure 3:**
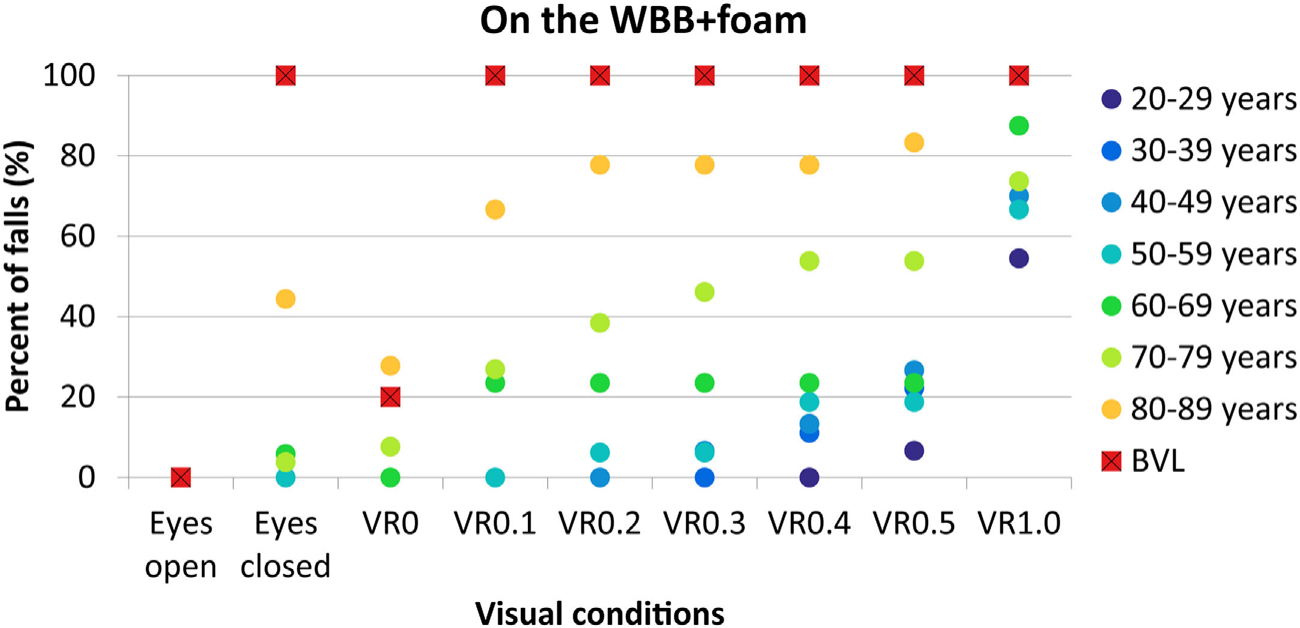
**Percentage of falls on the WBB plus foam in function of visual conditions for each decade of age from 20–29 years (dark blue circles) to 80–89 years (yellow circles) and for BVL patients (red squares with black cross)**. WBB, Wii Balance Board; VR, virtual reality; BVL, bilateral vestibular loss.

Percentages of falls per decade and for BVL patients on the WBB + foam for each condition are given in Figure 3.

No falls were observed with eyes open for each group of age and for BVL patients.

**Figure 4:**
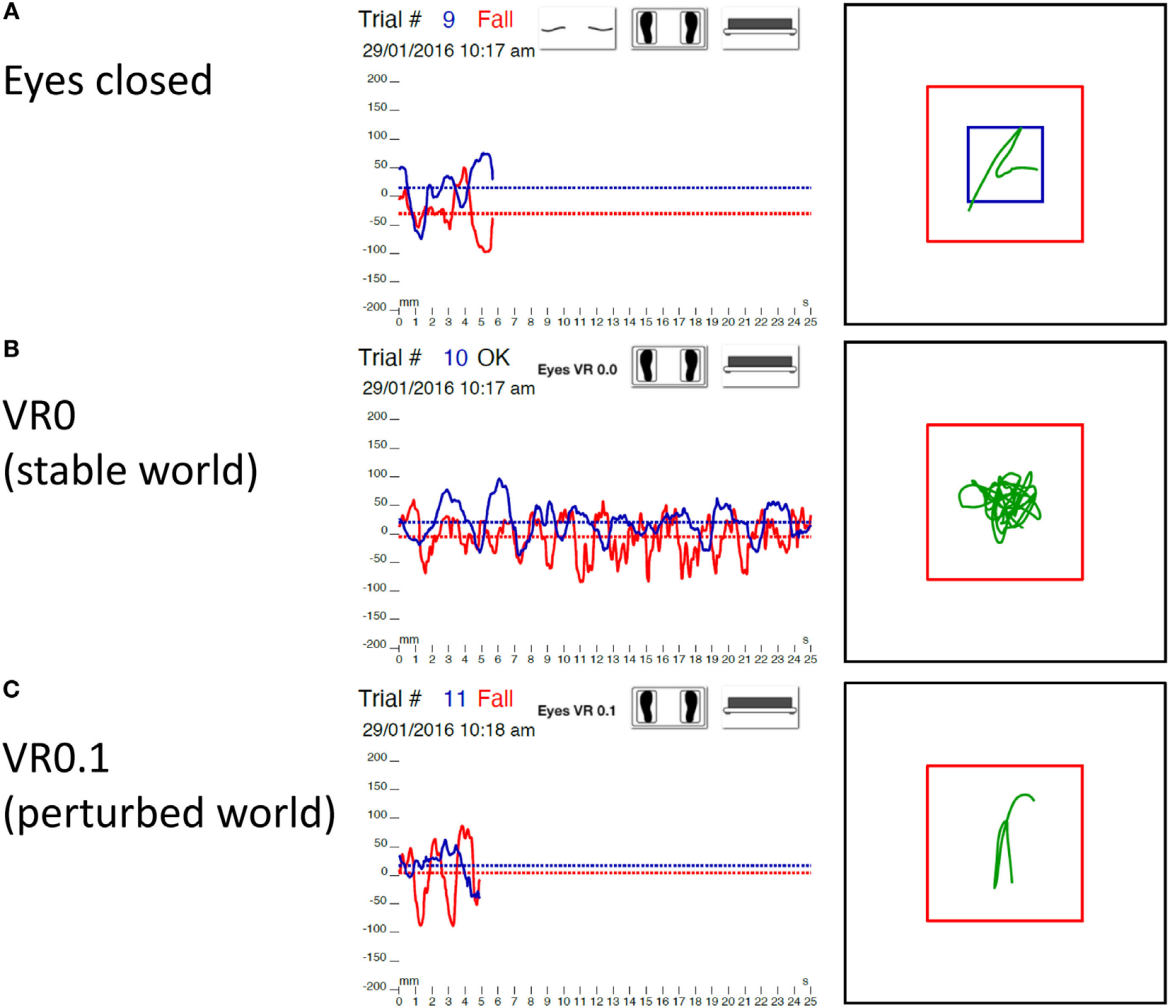
**Statokinesigram of a BVL patient on the WBB + foam eyes closed (A), with visual perturbation at VR0 [stable world (B)], and with visual perturbation at VR0.1 [perturbed world (C)]**. Right graphs: X (red trace) and Y (blue trace) trajectory of the center of gravity of the body in function of time. Left graphs: XY trajectory of the center of gravity of the body (green trace) with the limits of normality (blue square) and limits of stability (red square). Noticed that this BVL patient failed on the WBB + foam with eyes closed and with visual perturbation at VR0.1 but did not fall with a stable visual world (VR0). BVL, bilateral vestibular loss; VR, virtual reality; WBB, Wii Balance Board.

No falls were observed with eyes closed below 60 years. Few falls were recorded in decade 60–69 years (5%) and in decade 70–79 years (3%). Forty-four percent of subjects in decade 80–89 years failed and 100% for BVL patients (Figure 4A).

At VR0, no subject failed below 70 years. Few falls (7%) were observed in the decade 70–79 years. Twenty-seven percent of the decade 80–89 years and 20% of BVL patients failed (Figure 4B).

At VR0.1, no falls were observed below 60 years old. Twenty-three percent of subjects failed in the decade 60–69 years, 26% in the decade 70–79 years, 66% in the decade 80–89 years, and 100% in BVL group (Figure 4C).

The percentage of falls for the higher amplitudes of perturbation is given in Figure 3.

### Young vs Senior Groups

**Figure 5:**
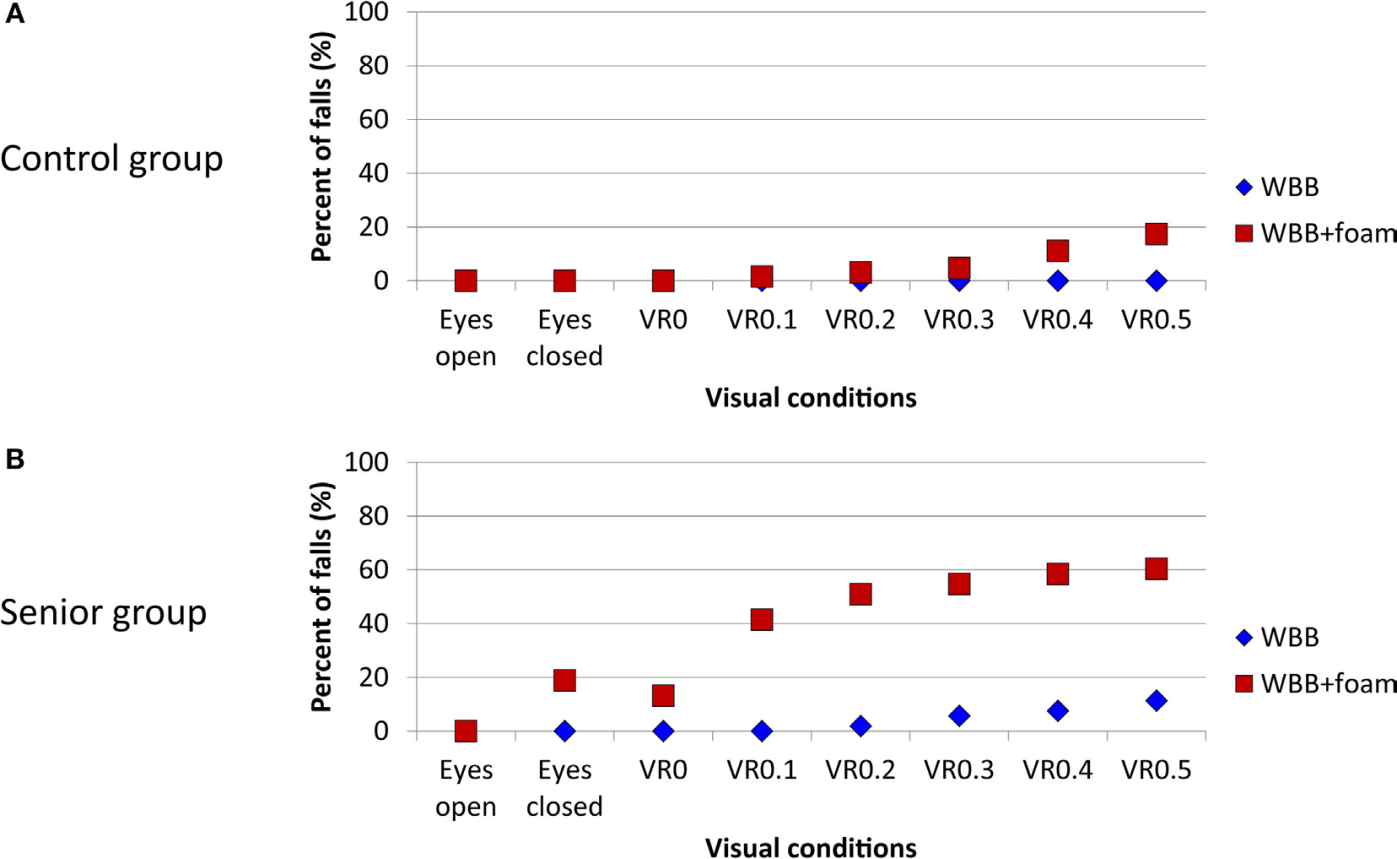
**Percentage of falls on WBB (blue diamond) and on WBB + foam (red square) for each visual condition in young control group (A) and senior group (B)**. WBB, Wii Balance Board; VR, virtual reality.

To be useful for clinical practice, we also compared the percentage of falls between young (control group, Figure 5A) and senior subjects (Figure 5B). Results with VR1.0 are not shown because, as previously described, this amplitude of the visual perturbation is too high (so that even young subjects failed) and so is not useful in clinical assessment.

### Comparison between Eyes Closed and Visual Perturbation at VR0.5

**Figure 6:**
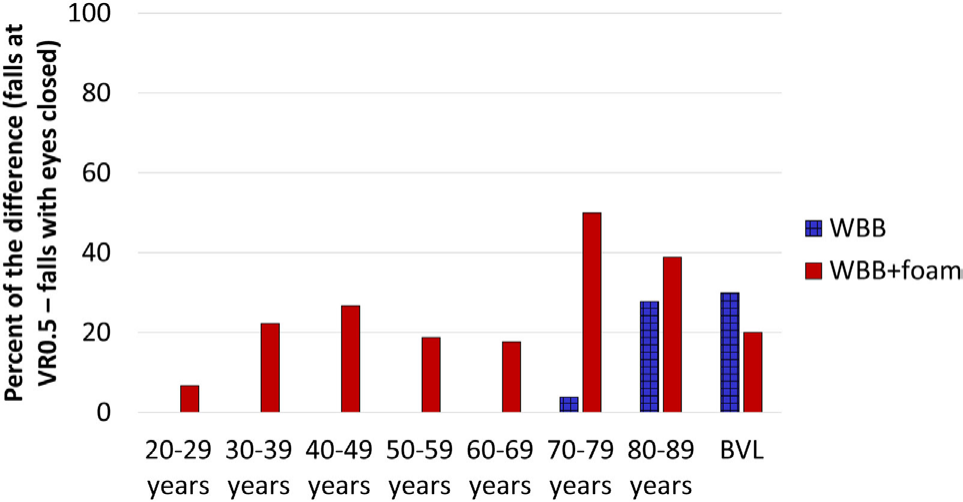
**Percentage of subjects who maintained balance with eyes closed but failed at VR0.5 for each decade of age and for BVL patients on the WBB (blue bars) and on the WBB + foam (red bars)**. VR, virtual reality; BVL, bilateral vestibular loss; WBB, Wii Balance Board.

Finally, we compared the percentage of falls between eyes closed and VR0.5. Results, on the WBB, showed that VR0.5 induced 30% more falls than eyes closed for the decade 80–89 years and for BVL patients (Figure 6). On the WBB + foam, VR0.5 induced more falls for each decade and for BVL patients. For example, in the decade 70–79 years, VR0.5 induced 50% of falls more than eyes closed. This suggests that had we relied on eyes closed, we would have missed 50% of subjects between 70 and 79 years who could be at risk of falls.

## Discussion

In healthy subjects, we found that the percentage of falls increased with age and with the amplitude of perturbation for both conditions: WBB or WBB + foam. The maximal amplitude (VR1.0) of visual perturbation used here is probably too high, since even in healthy young subjects in the decade 20–29 (the youngest group), we observed 9% of falls on the WBB and 54% on the WBB + foam. Moreover, patients (especially seniors) reported discomfort at this highest perturbation. This amplitude of perturbation should not be used in clinical practice. The other amplitudes of perturbation did not induce falls on the WBB for subjects aged between 20 and 79 years. For subjects older than 80 years, amplitudes of visual perturbation between VR0.2 and VR0.5 could be informative on the WBB (without foam) for detecting subjects with poor proprioceptive inputs for maintaining balance (they apparently rely more on their visual inputs than on their “stable” proprioceptive inputs) (8). In case of falls on WBB with visual perturbation, complementary tests are required to exclude medical problem (for example, vestibular tests, inferior’s limb electromyogram, ophthalmic tests), and rehabilitation should be done to prevent fall (9, 10).

Results on the WBB + foam are more scattered with increasing age. Indeed, for healthy subjects, we started to observe falls at different amplitudes of visual perturbation for each decade of age: some subjects failed at VR0.5 in the decade 20–29 years, at VR0.4 in the decade 30–39 years, at VR0.3 in the decade 40–49 years, at VR0.2 for the decade 50–59 years, at VR0.1 for the decade 60–69 years, and at VR0 for the decade 70–79 years and 80–89 years. Then, for each decade, the percentage of falls increased with the amplitude of the visual perturbation. This is congruent with the fact that with age, visual inputs became predominant in balance control (11–13), particularly in the case of unstable proprioceptive inputs (14). It could also be related to the increase of mental load involve in balance in seniors (15, 16). Indeed, the attentional demands specific to the postural control depend on cognitive factors, and mental load is correlated to falls (17). With age, seniors became less confident with their balance and have to think about it to avoid falls. The visual perturbation used in this study created a situation of sensory conflict with wrong visual inputs. So our visual perturbation is used as a distractor (18) that subjects needed to ignore to maintain their balance. Seniors may not be able to ignore the distractor because the mental resource is already involved in maintaining balance. Moreover, the mental load increased with the amplitude of perturbation because subjects needed to allocate more attentional demands for ignoring the visual perturbation. Finally, these results in seniors could be explained by the aged effect on the vestibular system (19). The canal function assessed by the vHIT did not change with age for the horizontal and anterior canal and slightly decreased for the posterior canal (20, 21). The otolith function assessed by VEMPs decreased with age, and seniors became non-responders (no VEMPs from both ears) to air-conducted and bone-conducted stimulation (22–25). For the senior group included in this study, we did not find any relationship between the percentage of VEMP non-responder and falls on the WBB + foam at VR 0.1.

For BVL patients, on the WBB, few patients (30%) failed with a visual perturbation between VR0.3 and VR0.5, probably because proprioceptive inputs compensated for the absence of vestibular inputs and for perturbed visual inputs (26). On the WBB + foam, all of them failed without visual inputs (eyes closed) and with perturbed visual world even at the minimal amplitude of perturbation (VR0.1) but they succeeded to maintain balance with a stable visual world (VR0) (Figure 4). We needed to reduce the amplitude of perturbation to determine the threshold of falls for BVL patients. This could be explained by the fact that, in these conditions, proprioceptive inputs are non-stable (by the foam) and visual inputs are perturbed (with VR) or absent (with eyes closed); and without vestibular inputs (BVL), subjects cannot maintain balance (27, 28).

We observed that stable visual inputs (VR0) induced fewer falls than eyes closed (without any visual inputs) whatever the subject’s group (healthy or BVL) and whatever the age decade. This is congruent with the fact that vision is the main sensory input at the central integration level to maintain balance (29). Moreover, using the VR for creating a visual perturbation at several amplitudes, we can assess balance performance more accurately than with the either baseline conditions eyes open and eyes closed. Indeed, since the visual perturbation at VR0.2 is smaller than the perturbation at VR0.5, it seems reasonable to hypothesize that the subjects who failed at VR0.2 are at higher risk of falls in everyday life than subjects who failed at VR0.5. This hypothesis could be tested by a prospective follow-up study: measuring balance with visual perturbations in a large number of subjects across all age ranges and after 1-year follow-up by assessing who actually had falls in real life. This relates the VR balance measure to the actual fall event and so would validate the VR balance measure as a predictor of the risk of falling.

**Figure 7:**
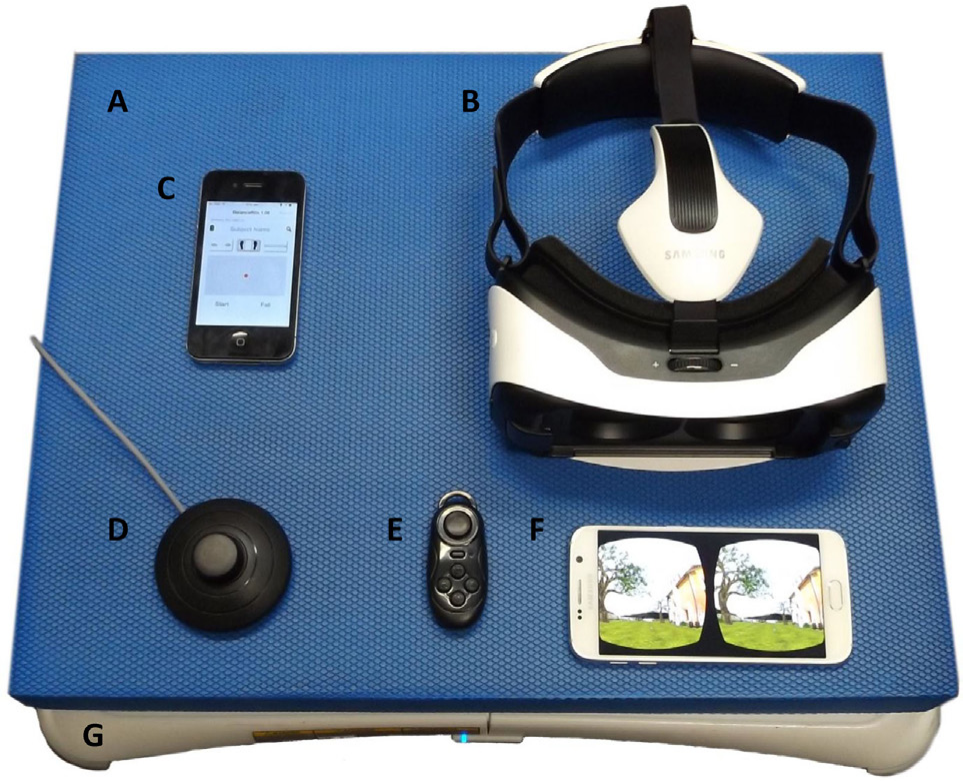
**New portable and mobile configuration of the material used in this study: (A) foam rubber; (B) Samsung virtual reality (VR) headset; (C) iPhone running BalanceRite App; (D) switch on/off foot button for the Wii Balance Board (WBB); (E) remote controller to launch the visual perturbation; (F) Samsung Galaxy S6 phone running TuscanVilla App (visual perturbation); (G) WBB**.

In conclusion, VR allowed us to develop this realistic new tool with a wide range of visual perturbations: rather than only two levels of visual condition (eyes open and eyes closed), the VR stimulus can be continuously adjusted to produce a visual perturbation that is anywhere between eyes open and eyes closed or worse than eyes closed. For example, subjects for whom the eyes open condition is too easy but the eyes closed condition is too difficult can be challenged or pushed to the limits of their ability by a visual perturbation that is effectively somewhere between eyes open and eyes closed. With this new tool, we can define a threshold for falls of each age group and patients. More interestingly, we can assess a threshold of falls for each individual subject and so evaluate precisely an effect of treatment or rehabilitation for each individual patient. This would allow the method to be used as a personalized medicine tool. Finally, the visual perturbation used in this study was projected into an Oculus Rift linked to a computer. Today, we are using the same visual perturbation but projected into a Samsung VR Gear (Figure 7) without any computer. This configuration allows the clinician to be mobile and to test patients anywhere from a regular consultation to the bedside. But it also could be used as part as a rehabilitation program by a physio or by a patient him/herself at home.

## Author Contributions

EC tested subjects, conducted the analyses and wrote much of the paper; WW tested subjects and conducted the data processing; SR wrote programs for the visual displays and for the data recording; HM developed the methods; IC wrote some of the paper and consulted; and CW devised the protocol and wrote some of the paper and consulted.

## Conflict of Interest Statement

HM and IC are unpaid consultants to GN Otometrics. The remaining co-authors declare that the research was conducted in the absence of any commercial or financial relationships that could be construed as a potential conflict of interest.

## Supplementary Material

Video S1 **Mixed reality video showing a subject on the Wii Balance Board (WBB) in the TuscanVilla perturbed world**. The perturbation used was VR0.5. The right bottom of the video shows, in real time, the screen capture of the iPod Touch recording the data from the WBB.

## Abbreviations

BVL, bilateral vestibular loss; ° or deg, degrees; EMG, electromyogram; VEMPs, vestibular evoked myogenic potentials; vHIT, video head impulse test; VR, virtual reality; WBB, Wii balance board.
